# Chronically Traumatized Children’s Ability and Willingness to Engage in Trauma Therapy: Experiences with The Sleeping Dogs Method

**DOI:** 10.1007/s40653-026-00872-9

**Published:** 2026-04-18

**Authors:** Sarah B. Schoorl, Marc J. Noom, Bregtje van Elven, Karlijn F. Andeweg, Danny J.C. de Bakker, Olaf Goorden, Suzan Polet, Heleen I. J. Sillekens, Eveline van Vugt, Geert-Jan Stams, Ramon J. L. Lindauer

**Affiliations:** 1 Department Child and Adolescent Psychiatry, University of Amsterdam, Amsterdam, and Levvel, Academic Centre for Child and Adolescent Psychiatry, Amsterdam, The Netherlands; 2 Department of Child Development and Education, University of Amsterdam, Amsterdam, The Netherlands; 3 HSK Groep, Amsterdam, The Netherlands; 4 Stichting Jeugdformaat, Rijswijk, The Netherlands; 5 GGZ, Breburg, The Netherlands; 6 iHub Education and Family Services, Rotterdam, The Netherlands; 7 SvPO, Amsterdam, The Netherlands; 8 Sterk Huis, Goirle, The Netherlands

**Keywords:** Trauma treatment, Chronic PTSD, Children, Stabilization, EMDR, TF-CBT

## Abstract

Engaging chronically traumatized children in treatment is often challenging, as they frequently have a history of failed interventions and significant behavioral issues. The Sleeping Dogs method, a specialized approach for preparing these children for trauma treatment, focuses on stabilizing and building their intrinsic motivation to engage in treatment. The aim of the present study was to examine the Sleeping Dogs method by interviewing 16 children and their practitioners about helpful factors in fostering willingness to discuss traumatic experiences and engaging in trauma treatment. Results suggest that the Sleeping Dogs method is effective in helping children to become both willing and able to talk about their traumatic experiences and engage in trauma treatment. Children and practitioners especially valued the Sleeping Dogs method’s proactive and motivational approach with its focus on autonomy and the vital role of supportive adults in the child’s environment. Future studies should examine implementation processes, adherence factors, and the method’s efficacy across various child profiles.

## Introduction

Chronic traumatization, particularly in childhood, has been proposed to result in a complex of symptoms that includes posttraumatic stress disorder (PTSD) (Cloitre et al., 2009). These children are often being exposed to multiple forms of traumatic experiences with an interpersonal and developmental character, such as child maltreatment and/or abuse within the family (Hiller et al., 2016; Van der Kolk et al., 2009). Unlike single-case trauma, these children do not tend to recover easily from these events, without therapy (Pellemans et al., 2023; Van der Kolk, 2021). Untreated chronic traumatization can lead to increased symptom severity, as well as a wide range of health consequences and negative consequences for the child’s development, such as school delays (Felitti, 2017; Howgego et al., 2005). Additionally, it can result in comorbid behavioral problems, including anxiety and depressive symptoms, attachment problems, and delinquent behavior (Fox et al., 2015; Renn, 2002) which persist into adulthood (D’Andrea et al., 2012; Van Vugt et al., 2014). While trauma treatment is recommended, engaging chronically traumatized children in therapy is often challenging, as they frequently have a history of failed interventions and significant behavioral issues (Struik, 2019). Therefore, it is necessary to prepare and stabilize them for treatment (Kliethermes et al., 2014). However, there is a lack of guidelines on how to prepare these children to engage in trauma treatment. In this article, we evaluated the Sleeping Dogs method, an approach designed to prepare and engage chronically traumatized children for trauma treatment.

###  Resistance and Motivation in Trauma Treatment

Trauma treatments, such as eye movement desensitization and reprocessing (EMDR) and trauma focused cognitive behavior therapy (TF-CBT), can reduce trauma-related behaviors, and are therefore recommended for children with traumatic symptoms (Greenwald et al., 2012; Hoogsteder et al., 2022; de Roos et al., 2017). EMDR involves a trained practitioner guiding the client through a structured process which includes recalling emotionally distressing memories while simultaneously focusing on a distracting stimulus. This approach activates the persons natural processing mechanisms, allowing the memory to be integrated and remembered rather than re-experienced (Shapiro, 2018). TF-CBT focuses on cognitive-behavioral and family system principles, emphasizing the ability to reflect on, understand, and modify maladaptive trauma-related thoughts, emotions, and behaviors. The practitioner will learn and help the child in re-regulating their trauma responses and master avoidance of trauma reminders and memories. To do this, the child and the parents need to express their personal thoughts and feelings about the child’s trauma experiences (Cohen et al., 2016). Therefore, initiating trauma-focused therapies requires children to recall and discuss their traumatic experiences.

For some children with chronic trauma, however thinking or talking about their trauma can easily result in anger, distress, dissociation or other negative feelings and behaviors. To protect themselves, chronically traumatized children may therefore try to avoid confrontation with their traumatic history (Greenwald et al., 2012) and may be reluctant to talk about their traumatic experiences to others (Farkas et al., 2010; Struik et al., 2017). The child’s barriers to trauma treatment are usually caused by a combination of their (unstable) current circumstances, the nature of the traumatizing events and their thoughts and feelings related to the traumatic experiences (Greenwald et al., 2012; Struik, 2019;). Furthermore, common for this population is that they already have had negative experiences with mental health services, which makes them even more resistant to therapeutic help (Greenwald, 2005). In addition, clinicians and parents may fear that confronting the child to talk about their trauma will dysregulate the child, and increase their problem behavior and mental state before symptoms reduce. A common belief is that children first need to be considered “stable” and “ready” before they are eligible for trauma treatment focused on trauma processing. However, without professional support, these children may never feel “ready” to talk about their traumatic memories, leaving their symptoms untreated (Struik, 2019).

### The Sleeping Dogs Method

The Sleeping Dogs method is developed for children who are initially not able or willing to engage in trauma treatment and is based on the idea that when children will not come to therapy, therapy should come to them. The term “sleeping dogs” refers to the traumatic memories that the child is either unwilling or unable to disclose, discuss or reprocess. Confronting these memories often leads to heightened anxiety and can exacerbate their distress, leading the network and caregivers to prefer to “let sleeping dogs lie’’. In addition, these children often feel incompetent, helpless, and disconnected from their network. Their basic psychological needs - which are competence, autonomy, and relatedness - are not fulfilled, and according to the self-determination theory (SDT) these three constructs guide intrinsic motivation (Ryan & Deci, 2000). The Sleeping Dogs method offers a structured framework for treatment by focusing on strengthening these psychological needs and by removing their barriers. The underlying premise is that when a child’s sense of competence, autonomy, and relatedness is enhanced, their motivation to engage in trauma treatment will increase.

Practitioners support this process by implementing targeted interventions that address these needs. Interventions that strengthen competence typically involve psychoeducation aimed at helping the child better understand how trauma has impacted them, and how therapy can help them overcome those difficulties. Interventions aimed at enhancing autonomy focus on encouraging the child to take an active role in decisions regarding their treatment and by providing the child with opportunities and tools to regulate their own behavior and emotions. Relatedness is strengthened by actively involving the child’s family and network and mobilizing them to support and motivate the child, provide their views on the child’s responsibility for the trauma, and if applicable explain why they have harmed or not protected the child. The emphasis on fostering intrinsic motivation aligns closely with the Self-Determination Theory (Ryan & Deci, 2000), which posits that interventions promoting safety and a sense of agency support autonomy, psychoeducation and skill-building enhance competence, and the involvement of the support network strengthens relatedness.

The first step of the Sleeping Dogs method is to identify the child’s barriers to trauma therapy, which helps the practitioner to determine the specific areas that require attention in treatment. A review into resistance of chronically traumatized young people to engage in trauma focused therapy led to five underlying factors (barriers) (Struik et al., 2017). The first barrier is related to feelings of safety and refers to children who may be reluctant to discuss their traumatic experiences due to a sense of (physical) unsafety. They are afraid of the consequences or do not have emotional consent from their parents to do so. The second barrier pertains to the multitude of challenges encountered in everyday life. Children experiencing this barrier lack the willingness or ability to talk, because of their lives being too unstable, or they fear that their life and routines will be disrupted because of going into therapy. The third barrier is the absence of an attachment figure or sufficient support from this figure. Children often do not dare to talk about their memories and problems in the family, because they are afraid of the responses of their parents, for example that discussing the trauma will upset them. They often cannot rely on their parents to support them, and they do not have anyone else to do support them. The fourth barrier is a lack of emotion regulation, and refers to children who do not want to talk about their experiences, because they are afraid to feel their emotions and fear that they will be unable to cope with them. Lastly, the fifth barrier is the cognitive shift. Because of the chronic nature of the trauma and the frequent involvement of parents, children can develop distorted or false cognitions about these memories. During trauma processing they need to realize they were innocent, and that their parents were responsible for the trauma they experienced. Children may experience guilt and shame about their experiences and fear their parents will reject them if they hold the parent accountable.

The second step is to develop an intervention plan to address the child’s barriers. This plan is customized for the child and can vary in time or type of sessions and type and number of interventions. Many interventions involve the child’s support system or child protection, and do not require the child’s involvement initially, allowing treatment to start without their immediate engagement. The interventions in the intervention plan can be delivered by people in the young person’s (professional) network who they trust or in whom they have confidence, such as (residential) caregivers, foster care workers, child protection workers, other professionals, or people from the child’s social network.

The Sleeping Dogs method is always combined with an evidence-based trauma treatment such as EMDR or TF-CBT, as the goal of Sleeping Dogs treatment is to engage them in and support them to complete such treatments. The layered approach ensures that stabilization is achieved progressively, allowing children to reach the necessary readiness for trauma processing at their own pace, providing chances for initial and gradual success, even if full stability is a longer-term goal. The treatment plan prioritizes interventions that target the child’s barriers, which should shorten the preparation phase to start trauma treatment (Struik, 2019). The Sleeping Dogs method then focusses not only on removing barriers for trauma treatment but provides interventions to support completing EMDR or TF-CBT and to prevent dropout, which also improves the child’s positive development and overall well-being.

### Pilot Study

The potential effects of the Sleeping Dogs method have been examined in a pilot study (Struik et al., 2017). A total of 14 children were included in the study and received Sleeping Dogs and EMDR sessions. All children were identified as being stuck in their treatment process due to their behavioral problems. As soon as the participants were ready to talk about their traumatic memories, EMDR sessions began. The caregivers completed standardized questionnaires using the Trauma Symptom Checklist for Young Children (TSCYC; Briere, 2001) within four to six weeks before and after the EMDR sessions. After two months, the case manager provided the clinician with qualitative information about the child’s well-being. At the end of treatment, all children engaged in and completed trauma-related therapy, and lower means were found for symptom severity in the depression and intrusion clusters of the TSCYC. These results indicate that this method can enable children to talk about their traumatic memories, and thus make them eligible for trauma-related therapy. While this study found positive results and effects, it did not identify what changed the children’s willingness and motivation to enter therapy.

### The Present Study

In this qualitative study, we explored the mechanisms of change that contributed to preparing and motivating children towards trauma treatment. Mechanisms of change’ refers to specific therapeutic factors or interventions that fostered a child’s readiness, motivation, and engagement in trauma therapy. We investigated how the Sleeping Dogs method is evaluated by children and practitioners and their reflections on the ingredients that contributed to being willing and able to talk about their traumatic past and engage in treatment. Specifically, we investigated their reflections on which barriers were present, what the practitioners did to remove them, and what effect this had on the willingness and ability of children to talk about their traumatic past. Therefore, the research question was: ‘’What are the mechanisms of change of the Sleeping Dogs method according to the children and their practitioners?‘’.

## Methods

### Design

This study used a qualitative narrative approach to evaluate the mechanisms of the Sleeping Dogs method. Seven Dutch youth residential care centers participated in the study, all of which served children with complex trauma. For this study, we interviewed 16 children and their practitioners. Since some children were treated by the same practitioner, some practitioners were involved in multiple interviews. This research design was approved by the Ethical Review Board of (blinded).

### Participants

The children (*n* = 16) all showed trauma-related symptoms and were initially unwilling or unable to engage in trauma therapy. Ten were female and six were male. At the time of the interview, their ages ranged from 11 to 21 years, while during treatment their ages ranged from 9 to 20 years. All had experienced chronic, mainly interpersonal trauma, including child maltreatment or domestic violence. Almost all of the children had previously received (unsuccessful) care. Each child received the Sleeping Dogs method to support engagement with EMDR or TF-CBT, and to sustain motivation throughout and after treatment. The duration of treatment including EMDR varied across participants, ranging from a few months to several years. This variation was considerable, as many were still receiving other forms of treatment at the time of the interview or re-entered care when new traumatic memories emerged.

The practitioners (*n* = 12) were all trained in the Sleeping Dogs method, at a minimum of Sleeping Dogs Trauma Specialist level III. All identified as female, with ages ranging from 30 to 60 years. All held at least a bachelor’s degree. Clinical work experience ranged from 6 to 38 years, and specific experience with Sleeping Dogs ranged from 2 to 11 years. The number of children treated per practitioner varied from 5 to approximately 100. Among those working with children in out-of-home care, both residential and foster care teams were required to implement the method. These teams also completed Sleeping Dogs training level III as a group, rather than individually, to ensure consistent team-based application.

### The Sleeping Dogs Method

There are two checks, as described in the Sleeping Dogs tool, to assess whether a young person is able to engage in trauma treatment. The first check is whether they are willing to talk about their traumatic experiences: if they agree to engage and can explain how they will benefit from processing their trauma, they have sufficient motivation and pass the motivation check. The second check, the nutshell check, is whether they are able to reprocess their traumatic experiences: they are asked to provide a very brief summary, in a nutshell, of the traumatic memories they want to work on. If they can do that without becoming too dysregulated or overwhelmed, they also pass this check. These checks can be done by clinicians, but also by caregivers. If they fail at one or both checks or even refuse to do the checks or come to therapy, they are suitable for Sleeping Dogs.

Secondly, the practitioner investigates the child’s barriers and develops a plan to target the identified barriers and engage the child in evidence-based trauma treatment as soon as possible. The practitioner reports this in the Sleeping Dogs tool (see: https://www.ariannestruik.com/wp-content/uploads/2023/10/9780367076146_E-resource-1-Barrier-Tool-colour.pdf), consisting of a case conceptualization, barrier form and an action plan. In the case conceptualization the practitioner provides an overview of the young person’s symptoms, traumas, timeline, the complicating factors and their network. Subsequently the practitioner, together with other professionals involved, fills in the barrier form and answers the 19 yes/no questions about potential barriers for the young person to engage. These are clustered around five themes (the barriers): safety, daily life, attachment, emotion regulation and cognitive shift. Based on these hypotheses about barriers, the practitioner plans interventions to overcome those barriers in the action plan part of the Sleeping Dogs tool. More information on the specifics of the Sleeping Dogs method and the tool can be found in the Sleeping Dogs Handbook (Struik, 2019).

Before implementing Sleeping Dogs, the organization assesses what prerequisites are needed, such as sufficient referral options for trauma therapy, collaboration with parents, coaching of group leaders in the method, handling transference and countertransference, and supervision for practitioners and psychologists. After the implementation is completed, organizations develop a plan for ongoing training and education of new staff members. Additionally, designated staff members within the organization periodically organize booster sessions to refresh the methodology. An implementation process typically takes 2–3 years to complete. The implementation of the Sleeping Dogs method includes a two-day workshop, three or four team supervision sessions for the residential caregivers lasting two hours each, and a follow-up day. Practitioners are trained to at least Sleeping Dogs Trainer Level III through completing the Sleeping Dogs Level II training, and competency-based supervision, requiring a minimum of eight hours and submission of five completed Sleeping Dogs tools. Additionally, there is the option for the social workers who work with the parents to complete a two-day Sleeping Dogs Level II training, along with a one-day workshop on making trauma healing stories followed by 10 group supervision sessions, each lasting 30 min, and making five trauma healing stories.

### Procedure

The researcher sent an email to all known Sleeping Dogs practitioners from Dutch organizations that have implemented the method up to level III. In the email practitioners were asked if there were chronically traumatized children in their caseload who initially were unwilling or unable to engage in EMDR or TF-CBT, but completed EMDR or TF-CBT after receiving the Sleeping Dogs method and were open for an interview. The main reason participants declined the interview was that revisiting the period when they were struggling felt too upsetting. All eligible participants were first approached by a member of the mental health organization (often their practitioner) and when interested in participating in our study, introduced to the researcher. The researcher would then explain the aim and procedure of the interview and made an appointment for the interviews. The interviews were carried out with the children and their practitioners separately. Interviews were held between April 2023 and August 2024 by three researchers. Before the interview started, informed consent was obtained from the child and by one of the parents or the caregiver in case the child was under the age of 16. Consent was also obtained for the interviews with the practitioners. The informed consent form provided a description of the research and asked for the child’s and practitioner’s agreement to use their information for research purposes. It also informed them that they could withdraw from the study at any time. Interviews took around 30 to 60 min. Practitioners were also asked to fill out some background information about themselves and the child. After completion of the interview, children received a gift card of 20 euro for participating, practitioners received a gift card of 25 euros for participating. The interviews were audiotaped, and afterwards literally transcribed.

### Interview

The interviews were either held at the residence of the child, the care center of their treatment, or online. The guideline of the interview was the same for both the children and the practitioners. The interviews followed a semi-structured interview guideline, in which five topics were listed beforehand: lack of safety, too many problems in daily life, not enough support and attachment, disturbed emotion regulation, and being stuck in negative trauma related cognitions. During the interview all barriers were explained, the children and practitioners were asked with each barrier whether they believed this was a reason for the child to initially not want to engage in trauma treatment. In case the barrier was present, the interviewer discussed which interventions the practitioners used to remove them and what was considered helpful for the child in becoming willing and able to talk about their traumatic memories and going into treatment. Using a semi-structured interview guide allowed us to ask follow up questions to explore answers in a more in-depth way. For example, when a child expressed that their parents reassured them the traumatic experience was not their fault, the interviewer asked about the child’s feelings regarding that reassurance, the reasons behind those feelings, and the impact (or consequences) those feelings had on them. The children described their own experiences, and what they remembered in hindsight as helpful, whereas practitioners described the interventions they provided for the children and their view on what was helpful. For the interview guideline see Appendix A.

### Data Analytic Method

The qualitative data were analyzed by the constant comparative method, as outlined by Kolb (2012). All interviews were imported and analyzed in MAXQDA using an iterative approach. First, open coding was conducted to identify relevant quotes related to barriers and interventions. These were examined inductively to develop initial concepts, categories, and patterns without imposing preconceived ideas. Next, axial coding was used to explore relationships between codes and to organize them into broader categories or themes. During this process, codes were reorganized or newly created to clarify underlying structures within the data. In the final stage, selective coding integrated and refined themes into a coherent analytical framework. Core categories were developed describing child-related barriers and mechanisms underlying the Sleeping Dogs method. A single participant response may include multiple statements that were coded into different categories. To ensure confidentiality, exemplar quotes are identified by a number of the participant.

Consistent with a constant comparative approach, we emphasized consensus-based coding rather than coefficient-based reliability. Initial coding was performed by the first author, followed by multiple iterative rounds jointly reviewed with the second author. In these sessions, interpretations were systematically compared, discrepancies discussed, and codes refined until consensus was reached. When disagreements remained, the final author provided an independent perspective. This iterative adjudication strengthened the rigor and consistency of the analysis.

Researcher reflexivity was addressed through ongoing reflection on the interviewer’s role as a researcher rather than a treating professional (Olmos-Vega et al., 2022). To support analytic validity, emerging interpretations and preliminary findings were regularly discussed with practicing clinicians to ensure clinical plausibility and contextual grounding.

## Results

**Fig. 1:**
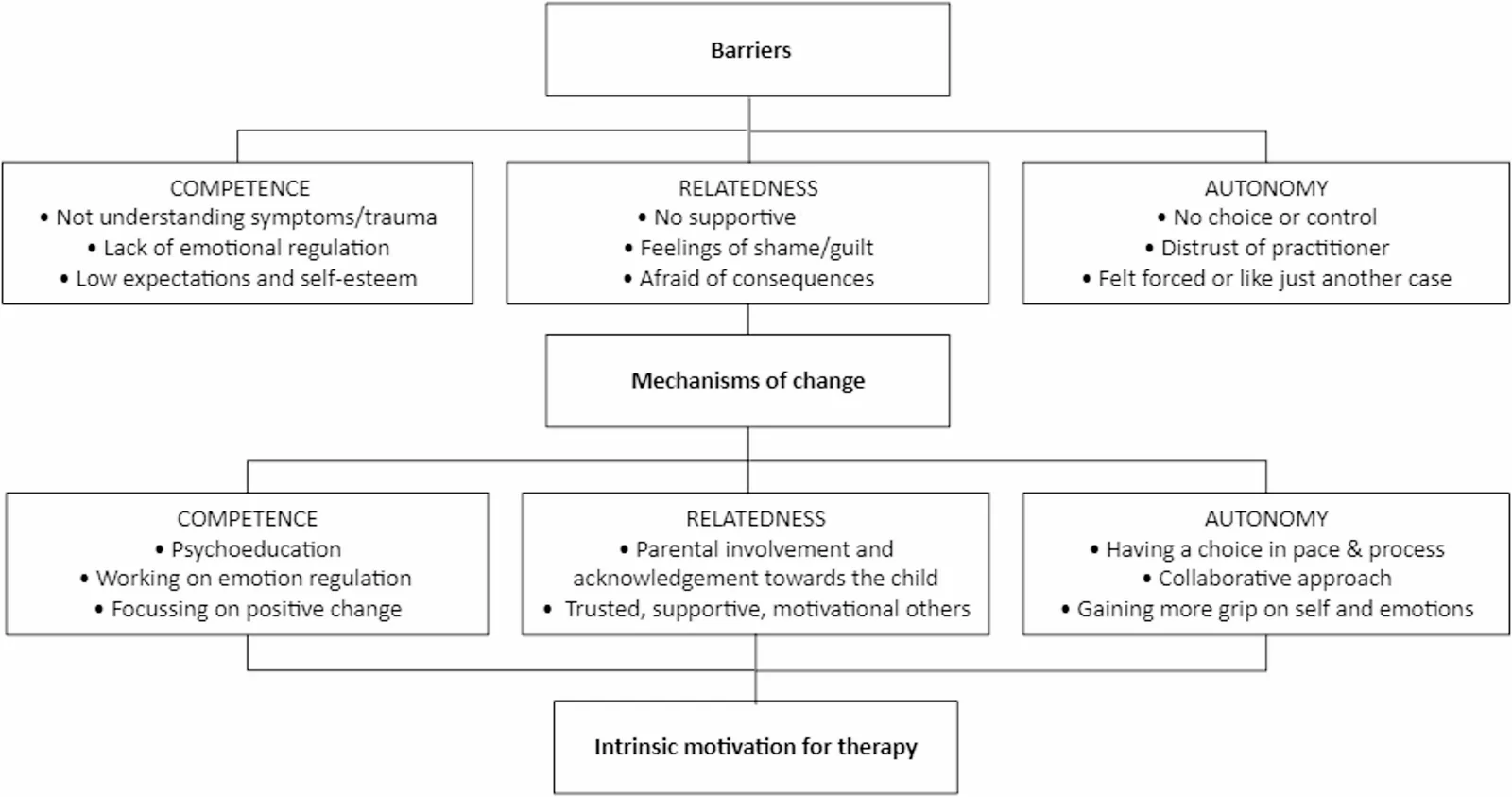
Conceptual model linking the SDT constructs with the identified barriers and mechanisms of change of the Sleeping Dogs method

We explored the mechanisms of change that children, initially hesitant to discuss their trauma, found helpful in becoming willing and able to talk about their experiences and engage in trauma treatment. This was based on interviews conducted with both the children and their practitioners. First the child and practitioners views on the barriers that were present in the children are reported. Next, the child and practitioners views on the mechanisms that were related to making the child willing and able for trauma-therapy are reported. Figure 1 presents a conceptual summary of the main findings.

### Barriers

All children reported that they did not feel safe discussing their memories or experiences. Some children were afraid that there would be consequences to being open about their feelings and experiences, such as that they would have to leave their (foster) homes or that they could not see their parents anymore. Many children did not want to talk about their traumatic memories, because they were afraid of the responses of others, or to burden others. Some children were afraid that their others, often their parents, would not understand them or reject them: ‘’*Let me think. I think for me*,* the issue was more that*,* as a child*,* I was never really believed. I wasn’t really taken seriously’’).* (C7 aged 18). It could also be that children did not have people around to support them or they were afraid their parents would get upset when they would discuss their feelings towards them: *‘’There were a lot of things that couldn’t be discussed because my parents have strong emotions’’).* (C6 aged 18). These fears were frequently connected to the belief that the child was responsible for the situation and/or did not deserve treatment: *" I always blamed myself*,* I would hear from my mom that… I was doing it wrong. I was a hyperactive boy and a problem child.‘’*). (C3 aged 18). Some of the children were afraid of the emotions that would arise within themselves when talking about the traumatic experience and therefore they tried to avoid talking. Furthermore, children were afraid of the impact that talking about the trauma, and going to therapy would have on maintaining their daily life, such as going to school:

Yes, I thought that there would arise a lot of emotions, and that I would perform even worse because of that, at school for example. I thought I would become very emotional, so a reason for me not to go into therapy was that I was afraid to go through my emotions. (C5 aged 15)

### Mechanisms of Change

#### Reassurance of Safety and Consequences

First, most children felt reassured when their practitioner emphasized the privacy of therapy sessions, knowing their conversations would remain confidential and would not lead to negative consequences or punishments, helping them open up more comfortably:

There was, at a certain point, a very clear distinction between me and the fact that she really knew she could confide in me (…) and, she noticed it too in our conversations that I didn’t share some things with her mother. You know, what she shared with me in confidence—I didn’t say anything about that. So, in that way, I earned her trust as well. (PR5)

This was often achieved by involving others, such as a parent or other significant person in the child’s life. In addition, sometimes judicial institutions like the child protection services or the child’s guardian were contacted, who could reassure the child that nothing would change and that contact with their parents would continue as usual:

What I did in that situation was simply maintain contact with the guardian, and the guardian emphasized the importance of maintaining those connections no matter what. They made it clear they would not change that. So, essentially, the intervention was a conversation to clearly communicate this. (PR8)

#### Explanation about Trauma

One of the interventions that was widely mentioned was the explanation of the relationship between the child’s daily life problems and their traumatic memories: ‘’*Together we put the puzzle*,* how her life has been and how those issues she was facing now are connected to the past’’* (PR6). These explanations and reflections gave children more insight and better understanding of where their behavior came from, especially when examples were specifically related to the situation of the child:

The focus was primarily on what she does and where that comes from, essentially trying to normalize everything. She blames herself for everything and feels responsible for all that has happened, thinking it’s her fault and that she deserves it. (…) Being able to consistently trace back to where these feelings originated has really helped her. (PR11)

Sometimes the practitioner constructed a trauma narrative with the child (and caregivers) to acknowledge the logic of experiencing the (often negative) feelings of the child. Many children felt at fault or at blame for their past, and experienced that they were responsible for their parents instead of the other way around. Explaining that these thoughts are common reactions to trauma and that they are not meant to be permanent often creates hope and a step towards a shift in beliefs. Furthermore, realizing that their experiences are not their fault and that they are not destined to be “bad” fostered a sense of empowerment in the children, and reduced their feelings of helplessness: ‘’*That was helping me realize that it’s okay to feel bad. It’s okay to be depressed. It’s not your fault; it’s due to the upbringing you’ve received’’* (C1 aged 21). This understanding inspired children with a greater sense of control, hope, and motivation for change, and encouraged them to engage in conversations about their experiences. In all cases, unless not possible, this narrative story was constructed together with the child’s parent(s):

We first started by acknowledging these through the drawing she made with the practitioner, highlighting all her experiences and the behaviors we observed in her. We recognized together that neither of them could do anything about what had happened; it was something that had occurred to them. Given all these circumstances, they were actually doing really well. So, we began with that acknowledgement and explained where the outbursts came from and why things clashed between them. (PR5)

These explanations often yielded to more understanding and self-worth in children (and caregivers), and therefore often more motivation to work on themselves.

#### Motivation for Change

Moreover, practitioners motivated the children to work on themselves by focusing on what they want for the future, giving them control, and how they can change their behavior to do so, making them more capable in making choices. It was specifically valued by children that the focus was on changing the trauma-related behavior, and how therapy would help with that:

The moment that they, instead of pushing me, explained to me what could be different and how I could change my responses to certain situations differently, I thought yes, I actually want this to change. (…) That helped me to get motivated, like this is what I want to achieve, instead of what I must do. (C1 aged 21)

To promote this motivation, children appreciated a plan that outlined how to achieve this by setting specific goals to be worked on throughout the treatment:

That was particularly helpful because I had a clear view of my life, with specific points laid out and what needed to happen.(…) like, what do I need to process or what do I need to do? It was all written down, so that was nice for me. (C11 aged 17)

*S*etting future goals not only enhanced the child’s motivation but also helped them recognize their progress, encouraging them to open up more. Moreover, children noted that the practitioner encouraged and motivated them by highlighting their progress and the accomplishments they had already achieved:

At first, I found it very hard to let her in, but then she told me already ‘’You actually already did it, you are already there, and you made progress, do not underestimate yourself so much.‘’ She has a really nice perspective. (C11 aged 17)

#### Structuring and Identifying Trauma-related Memories and Feelings

Some children found it hard to remember their memories, or did not have a clear overview of all that happened to them. Some practitioners therefore began by creating a timeline with the children (and their caregivers) based on their child protection files to explore and organize the traumatic memories, helping to make the events within the family clearer and more structured: *‘’Often you see that it is a chaos of memories*,* when you draw a lifeline*,* this can order the brain and give insight into what happened to me. That helped to structure the memories’’ (PR6).* Children often better understood what happened to them, when and why. This made it easier to identify where the most intense trauma memories were, providing valuable insight for both the practitioner and the child. It also facilitated more chances to encourage openness in discussing and addressing those specific memories.

In some cases, the practitioner and the child revisited places linked to the traumatic experiences, to provide the adaptive information that the child is safe now and the trauma is in the past, as well as to confront the child with the feelings that come up while being exposed to these memories. Often, it became clearer what the child had experienced and how it affected them because they were better able to express their emotions and share their thoughts related to the trauma:

A very important moment was when our outreach worker took her for a day trip to where she used to live. During that visit, many stories surfaced (…) There were things that we were also unaware of, which she shared with our outreach worker (…) You can then continue to work with that in therapy’’). (PR12)

#### Making Problems in Daily Life Manageable

To make treatment more accessible and to promote children’s motivation, interventions that focused on helping children find stability in their daily routines were provided. For more than half of the children, interventions aimed at creating structure in their day-to-day lives were crucial in helping them feel more competent and more supported to engage in therapy. For some children, this involved a change in contact arrangements, or a temporary removal from their home environment to make the child feel safe and stable:

We chose this setting purely because of the structure and stability it provides. I’m the kind of person who really needs that, and they’ve given it to me here. (…) The stress in my head, which came from my home situation, has pretty much disappeared. (C3 aged 18)

In other cases, providing additional support for attending school or addressing issues with eating and sleeping patterns proved helpful in fostering a sense of stability, Practitioners also emphasize this point: ‘’*He was barely attending school*,* so we involved a support agency as an intervention that accompanied him to school twice a week*,* but also supported him with daily tasks at home*’’ (PR7). For some children, it was necessary to attend school less frequently to prioritize therapy. Several children mentioned that due to their symptoms, they were struggling academically and could not envision managing both therapy sessions and school at the same time. As a result, the practitioner initiated discussions with the school to explore the possibility of temporarily pausing or diminishing their engagement in education:

Yes, she had a lot of flashbacks and triggers. T. went regularly with her to school, and eventually, together with the school and all parties involved, like child protection services, they were able to agree on a temporary exemption from school, so she could focus more on her treatment process. (PR11)

#### Making Emotions and Feelings Manageable

To stimulate the child’s emotion regulation, children were given explanations and exercises about emotions to better deal with these emotions or dissociation in situations that they found hard. Many children experienced stress when thinking about the traumatic memories. Therefore, practitioners together with the child worked on creating calmness and relaxation (a safe place) within the child’s mind, often using ‘here-and-now’ exercises. Often children felt more secure because of this exercise, and less overwhelmed when thinking or talking about the trauma: ‘’*The calming effect and to stay in contact with me during the sessions were also key objectives in the initial phase*,* which helped her feel a greater sense of safety internally’’* (PR6). For some children, it helped to feel safer while talking when there were adjustments or distractions in the (therapy) environment that led to less intense emotions within the child:

I can’t sit still, not really. You might notice that. There was always something on the table, and I would join in and play with it, so to speak. That helped keep me a bit occupied, which made it easier for me to talk about things. So I was engaged with something else too. (C15 aged 19)

Another aspect that was considered helpful was that children learned to recognize their emotions and to have control over them by various exercises:

I had a kind of ball and they explained to me that I decide for myself when to let my emotions in or not, it is your choice to decide when you want to feel the emotions of your past or present or not. I worked a long time with this example, and it helped me, now I can allow my emotions, but I can also stop them when I want. (C13 aged 19)

Most practitioners incorporated a plan describing what the child can do when feeling triggered or when being exposed to traumatic memories, which the child could use him/herself, or share with others. This plan often led to better insight into how to handle triggering situations and feelings of control in these situations:

We discussed how you can improve your situation: what are you already doing, and what could you do better? (…) Making that plan gave him the feeling that he was taken seriously and that was very important for him, I think. That he felt that he was heard and also that he knew what he could do when he felt bad. (PR3)

#### Collaboration and Availability of and within the Team

To gain more trust and motivation within the child, it was helpful that practitioners reached out to others involved with the child. These children found it hard to repeatedly open up to everyone, therefore it was also valued when the practitioner got in contact consistently with the child’s support network, including family members, caregivers, case workers and others involved in the treatment process to let them know the process of how it is going and what needs to be worked on: *‘’At one point*,* she had to create a life line. She realized that some parts were more difficult to discuss. So*,* I had to talk with someone else from the care team*,* and that’s when we started to uncover more’’.* (C11 aged 17). This often led to less resistance within the child, and more understanding and support from others, which contributed to the child’s progress and willingness to engage:

My practitioner always kept my aunt informed about how things were going, how everything went. And yeah, actually, my residential care worker also had contact with my practitioner, so they discussed things together. That way, my support workers always knew what was going on, like which memories I had processed and what I was working on at the time, you know, those kinds of things. So, there were always connections between them. (C14 aged 18)

Practitioners also emphasize the point that some children did not have parents, or people close to them anymore. For some therefore the availability and the support from their practitioner, others in their care team or the residential team was very valuable for the child and he connection with them led to opening up and feeling supported:

The fact that the residential care workers were involved in treatment was for N. very good. He was a real talker, so he could always talk to people when something was wrong or when he felt sad, or unsure. His insecurity was a big thing for him, so that helped a lot, the support of his team. (P3)

For some children it was helpful to have someone to support them in (going towards) therapy, especially when the child did not have someone else in their life who was close to him or her. The availability of the care team as well as the residential team, especially during the sessions, gave them the motivation and strength to engage in therapy: *“And she would bring me to the EMDR practitioner every time*,* otherwise I wouldn’t go (…) it really helped that someone came along*,* no matter what*,* whether by bike or anything. Just having someone go with you”.* (C2 aged 19)

#### Acknowledgement and Motivation of Significant Others

What was mentioned as helpful during therapy was shifting the child’s negative thoughts or beliefs that were associated with their traumatic experiences. Many children initially believed that they were (partly) at fault for the trauma, and therefore did not dare to talk about these memories. One of the most mentioned interventions was the involvement and acknowledgement of important (supporting) people, and the relationship with them. Almost all practitioners report that they used interventions that involved an important person in the child’s life, such as the parent, extended family members, caregiver or the foster parents. Involving people involved in the child’s life whose opinion they valued, led at itself to more willingness and trust in children to work along:

A. said the only way to get in real contact with me is when you get in contact with her (mum). I think eventually there are some people you really want to receive the message from, and I think that this has been very helpful, because in this case her mom was the only one that counts. (PR1)

In addition, involving the parent led to opportunities to achieve acknowledgement from the parent to the child about the child’s innocence. Interventions ranged from giving psychoeducation or help to the parents, arranging family conversations and creating a trauma healing story: a simple, illustrated narrative of a child’s life and the parent’s view on the trauma, used to discuss previously unspoken issues. It helps abusive parents reframe their actions, process their own trauma, and reduce self-blame while supporting their child’s healing. Often this led to the ability of parents to acknowledge their child’s innocence regarding the abuse or neglect, and recognize it was not their fault:

Yes, she (mum) wrote a letter back then, I remember. And she also said that not everything is my fault. And that it’s good for me to go to therapy; she genuinely wanted that for me. Yeah, I found it nice to hear, actually. It felt like a weight lifted off my shoulders. (C5 aged 15)

Often when the child received acknowledgement and acceptance of important people surrounding him or her (and often involved in the memories), practitioners noticed a more open attitude towards help and talking about the trauma in the children:

We gave psychoeducation to the parents, and then they acknowledged N’s innocence (…) From N. we heard that he experienced it as a relief for him that his father could say to him that he made mistakes, that these should not have happened and that it is important for him to process these memories (…) I could see a laugh on N’s face, and he started to relax more. Also, you saw in the residential group home that he began to talk more about that trauma. (PR3)

In addition, for most children this also led to a more positive attitude, realistic beliefs, and feelings that they are worth something and deserve treatment. Therefore, this intervention led to more acknowledgement and motivation to engage in therapy.

#### Connectedness and Support from and within Significant Others

Some practitioners referred to increasing the availability and the bond between the child and an important figure in his/her life. This included giving the parent insight into the child’s situation, promoting the need to spend more time with the child and making concrete plans on how the family member could support the child. Therefore, a support plan is often developed to outline how parents can assist their child in situations where parents may struggle to address the trauma themselves or may find it distressing to discuss it directly. Often these interventions led to the child feeling better comforted and supported and more motivated to be open towards talking about their trauma and to go into therapy:

I was motivated to spend more time with my dad and to be more open towards him (…) I mean with my dad I felt safe, but I was still emotionally closed, and once I started to spend more time with him, I once actually started crying near him and he could comfort me. (C7 aged 18)

For some children, a stronger relationship between the important people around them helped foster a more open attitude towards discussing their experiences. Some children felt unsafe and found it difficult to speak about their experiences to others, such as their foster parents, because they also felt loyal to their parents, and they were worried they would get angry or would reject their parents for harming them:

During the supervised visits, a lot of attention was of course given to N’s feelings of safety. For a year, there were visits involving both the foster parents and the mother together, which was N’s wish. This arrangement worked really well for an entire year, and during that time, you could see N. thriving. Everyone was quite relaxed, and the atmosphere was very calm. (PR4)

Practitioners therefore worked on strengthening these bonds and encouraged open communication, reassuring the child that their concerns are understood, but will have no consequences on their likeness of someone:

Also, his foster parents, they understand more about the child’s and parents history now, and where they used to be negative towards his mother, they now can see that his mother always tried to do the best for him and now they see that the conversations about his mother are less loaded at home. (PR15)

#### Autonomy in the Therapy-process

For some children, having the ability to express their own wishes and preferences during treatment was particularly beneficial. Allowing them to take control over certain aspects of the therapy, such as deciding when they were ready to start processing traumatic memories, when they had enough for the day or choosing the type of therapy that suited them best, empowered them to engage more actively in the process:

I immediately told her, girl, if you don’t want this, we simply won’t do it. I mean, I’m definitely not going to force you into processing. I just want to help you in whatever way we can, to support you with the things you’re struggling with. (PR5)

This sense of autonomy helped create a more comfortable and personalized therapeutic experience, contributing to the child’s intrinsic motivation. More than half of the children and practitioners mentioned that the child liked to experience control in the sessions, such as experiencing control over the planning of sessions and implementation of the therapy sessions, such as when they want to talk about something or not or when they would like a break:

In the sessions he sometimes walked out of the room, he had enough of it, and then later he came again. But he could indicate to himself when it was enough for him for the day. He did not like the therapy he did with me, and the PMT (psychomotor therapy: a body-oriented therapeutic approach that uses movement and physical experiences to support emotional, cognitive, and social development.) he liked. I think that is okay, and he could indicate that for himself. He had a lot of control in the sessions. (PR7)

It was mentioned that it was helpful to customize treatment to fit the child’s specific needs and to look into what was helpful for them, therefore the practitioner needed listen to the child and incorporate suitable interventions and behavior:

Look this is not a girl with whom you can teach how to regulate her breathing, I mean she would do it because she is a polite girl, but she would not use it at home for herself you know. So, I had to explore with her ‘what will work for you’ and adjust to that. (PR5)

Often when the practitioners got to know the child and adjusted their behavior and interventions towards the child, this helped them build a relationship with the child and led to the child opening up: *“We really put in the effort as a care team. And instead of distancing ourselves when she was upset*,* which would make her angry*,* we were able to recognize that she needed more closeness in those moments”* (PR2). The children mentioned that during the sessions they felt in control, comfortable, helped and more open to disclosing their thoughts and feelings. In addition, they mentioned that they felt that the help that was provided for them suited them and contributed to opening up:

When I had just come here (residential unit), I was in the garden and I started to cry very loudly, and they (residential caregivers) heard me. But when they heard that I was almost done crying, then someone came to me. So, they came to me when I was calmer, because when you are high in your emotions then you’re worth nothing. Then you can’t and don’t want to talk about it and you just want some rest. At least at those times I want to be left alone at first. (C3 aged 18)

## Discussion

The aim of this qualitative study was to identify the mechanisms of change that helped prepare and motivate children for trauma treatment. This was done by exploring the experiences of children who participated in the Sleeping Dogs method, through interviews conducted with both the children and their practitioners. By evaluating what led to motivation and engagement in trauma therapy in these children, it is core to the structure of the Sleeping Dogs method to first identify the underlying reason why the child hesitated or refused to engage in treatment or discuss their experiences. By addressing these core barriers, other obstacles often also became less important barriers or more easily managed. Children often had the feeling that they did not know how they could benefit from treatment, they did not understand their behaviors and the link to trauma, they could not self-regulate, they felt guilty, ashamed and like they were not important. In addition, they had the feeling that they were ‘just another case’, they were forced to talk or engage in treatment, or they did not experience support from anyone to motivate them to help them during treatment. These children did lack the self-determination needs of intrinsic motivation: competence, relatedness and autonomy (Ryan & Deci, 2000).

### External Factors Affecting Engagement in Trauma Therapy

First, to obtain a positive psychotherapeutic process, it is essential to prioritize the child’s sense of safety and stability (Norcross & Lambert, 2019). This was often facilitated by the practitioners attitude giving the child choice and control over treatment and providing them with information, as well as involving a trusted person of the child who could reassure the child that discussing their experiences would have no negative consequences. In addition, chronically traumatized children often face challenges in their home or school environments, leading to feelings of instability and insecurity (Topitzes et al., 2019; Vanderzee et al., 2019). This makes it difficult for them to prioritize and schedule treatment sessions, especially when their (foster) parents are also experiencing parenting-stress and cannot help them. When their routines are disrupted, engagement in treatment becomes even more challenging (Farrugia & Jos, 2021; State of Victoria, 2021). Therefore, but only when necessary, establishing a more stable daily routine could lead towards more engagement in treatment, which is commonly used in other therapeutic approaches (Myrick & Green, 2014). However, it is the focus on motivation and fostering autonomy, and the role of significant others herein, while working on these aspects that distinctly aligns with the Sleeping Dogs method. Compensating for the instability in the child’s daily life can motivate them and enable them to organize their routines in ways that support active engagement in therapy.

### Strengthening the Child’s Feelings of Competence

The results showed that children found it helpful to gain a better understanding of their behavior and symptoms, and the link to their traumatic experiences. Psychoeducation is a common component in various psychotherapies (Kooij et al., 2022), since it helps children to become aware of their symptoms and understand its origins (Greenwald et al., 2012). However, Sleeping Dogs psychoeducation differs from other psychoeducation as it not only provides understanding, but especially motivates for trauma therapy, and provides hope by explicitly explaining how therapy can help reduce symptoms and motivate for therapy. Explaining that their behavior can be changed seems especially important for children with chronic trauma, since this group often struggles to understand the benefits of discussing their traumatic memories, and shows avoidance (Greenwald, 2005). To effectively support children, it is essential to start by identifying and acknowledging their strengths and competencies. This approach lays the groundwork for positive change, especially in children facing challenges, and provides concrete starting points for treatment planning (Hodas, 2006). Moreover, realizing that and how they could change their behavior has been shown to enhance both the child’s competence and autonomy, since it gives them a sense of control and confidence (Cohen et al., 2010).

In addition, some children were afraid of (being overwhelmed by) their emotions, or becoming blocked by their emotions, when talking about their traumas. Therefore, teaching children to regulate their emotions can lead to feelings of being competent in talking about their memories, which may subsequently increase motivation for therapy. Focusing on experiencing more calmness and relaxation in the child’s mind and body can lead to the improvement of emotion regulation skills (Perry & Dobson, 2013) and feelings of safety and therefore more ability to talk about traumatic experiences. Some practitioners made a plan for the child to apply in daily life situations, helping children to better identify their trauma triggers and understand how to respond. This gave them the feeling that they were in control, which contributes to feelings of competence, as well as autonomy in the child (Cohen & Mannarino, 2011; Hodas, 2006). However, reassuring the child’s sense of safety and involving a trusted support figure often also resulted in significant improvements in their ability to regulate emotions. As a result, targeted emotion-regulation interventions became less of a priority in these cases.

### Strengthening the Child’s Connectedness (To Others and the Self)

Our results showed that one of the most important mechanisms of change was the involvement of the parents, who could alter the trauma-related cognitions within the child. Chronic traumatic experiences within the family can lead children to develop unhealthy thought patterns, including self-blame (Kerig & Swanson; 2010; Meiser-Stedman et al., 2019). Children often try to protect or care for their parents, and may avoid expressing their own distress to avoid upsetting them, which leads to distorted trauma-related thoughts, connected to the child’s relationship with their parents. As a result, parents can play a crucial role in offering new perspectives or understandings that help the child process its experiences in a healthier way (Mcllwaine et al., 2020). It has been shown that altering these dysfunctional trauma cognitions in children is one of the key drivers of reducing trauma symptoms (Woud et al., 2021). In almost all cases, there was a significant person that could acknowledge the trauma experiences of the child, and reassure them that it was not their fault. This led to feelings of recognition in the child, and less feelings of self-blame and shame, which contributed to more willingness and motivation to engage in processing traumatic memories and increased their ability to tolerate the trauma related emotions.

Moreover, parents can play a key role in repairing and strengthening the parent-child relationship (Bartels et al., 2019; Kiser et al., 2020). Our results showed that parents could not only acknowledge the child’s dysfunctional thoughts, but their involvement also contributed to more feelings of connectedness and support within the child. Involving the parents in the process and educating them about the child’s behavior, and the link to trauma and their behavior, can help them understand and also can boost the parents’ feelings of importance in the recovery of their child (Dwyer et al., 2024). In addition, creating a trauma healing story together with the child and the parent can improve the parents’ understanding of their child’s behavior and often even of their own behavior if they are traumatized too, and reduce the parent’s shame. This often facilitates them to motivate and support their child to engage in trauma therapy (Dwyer et al., 2024; Kooij et al., 2022), which seems especially helpful, since children tend to value their network support and opinion more than professionals support (Svensson, 2024).

In addition, the availability and support of the people involved in the child’s residential area and/or treatment process was acknowledged, such as the child’s residential carers, case managers and the clinical staff. Especially for children living in residential care, it is important to collaborate with the whole team of caregivers involved with the child, since they spend a lot of time with the child and know the child well. The caregivers can provide a secure base, optimize safety and support positive change within the child (Moore et al., 2018). Furthermore, they play an important role in motivating the child for- and supporting the child in therapy. In research of Dwyer and colleagues (2024), it has been shown that the Sleeping Dogs method can be implemented successfully in residential care, and can support practitioners wanting to enhance children’s access to and engagement in trauma treatment. It thereby supports the idea that children shouldn’t be expected to seek out therapy on their own; rather, therapy should be made accessible and brought to them.

### Strengthening the Child’s Feelings of Autonomy

Having a say and a sense of control over the therapy process was mentioned as a key element in motivating children to engage in therapy. Children with chronic trauma often lose trust in others (Greenwald et al., 2012), and therefore may be reluctant in listening and directly opening up towards the practitioner. However, Malchiodi and Crenshaw (2017) note that when children have difficulty verbalizing their problems, this leads to practitioners asking too many questions instead of allowing children to tell their own stories. Our results show that children want to be heard, believe they have a voice, and want to be treated as a unique individual requiring specialized treatment. The Sleeping Dogs method is customized as opposed to other stabilizing interventions, which makes it a better fit for children who are more complex in engaging in treatment (Struik, 2019). Moreover, the practitioner should obtain a caring, proactive and attentive attitude and ensure that the child’s voice be heard and valued (Moore et al., 2018). When children trust their practitioners, they are more likely to discuss their concerns, share information, and participate in collaborative decision-making, including engaging in therapy (Augsberger & Swenson, 2015). These feelings of autonomy in the therapy process increased children’s perception of choosing for themselves to engage in treatment.

### Limitations

While the results provide valuable insights into the factors that motivate children to discuss their traumatic experiences, and it is a strength that we included both the perspectives of children as well as their practitioner, several limitations must be acknowledged. First, the study sample consisted solely of children who benefited from the Sleeping Dogs method. These children successfully entered trauma therapy, and were afterwards able to reflect on their therapeutic experiences. As a result, the findings do not capture the perspectives of children who did not engage with or complete the Sleeping Dogs method, or those who were unwilling to discuss their experiences. However, since the focus was on uncovering mechanisms within successful interventions, this was not seen as a major disadvantage. Also, in some cases children and practitioners reflected on an intervention that was used, which, according the Sleeping Dogs analysis, was not necessary for engagement as the child did not have those specific barriers. Whereas these interventions could be valuable later in the treatment process, it is important to define the priorities for engaging the child in treatment to avoid unnecessary delay in the preparation phase (Greenwald et al., 2012).

The sample in this study is relatively small and homogenous, which limits the generalizability of the findings to other contexts. However, since the method primarily emphasizes fostering motivation for therapy rather than directly addressing symptoms, the results may still be relevant for children in different settings. Previous research underscores the critical role of motivation in achieving successful treatment outcomes across various conditions and cultural contexts (Self et al., 2023). Although effort was made to create a neutral interview environment, potential interviewer and social desirability biases should be acknowledged. We minimized interviewer bias since the interviews were conducted by independent researchers who were not involved in the participants’ clinical care. Furthermore, the use of predominantly open-ended questions minimized opportunities for steering or suggestive questioning. In a small number of interviews, the child’s practitioner was present. While this may have increased the likelihood of socially desirable responses, the practitioner’s presence also appeared to enhance participants’ sense of safety and willingness to share their experiences. We consider this a balanced trade-off, though it remains a relevant limitation to the interpretation of the findings.

#### Clinical Implications and Further Research

The results constitute a first step in substantiating the Sleeping Dogs method and can inform the development of trauma therapies, with the focus on strengthening the child’s competence instead of their problems. Instead of only referring their children to service providers, out of home care organizations can play a large role in increasing the child’s intrinsic motivation and engagement in trauma treatment with the Sleeping Dogs method, prior. This will increase the likelihood of successful completion (Annakin et al., 2025). Rather than waiting for a child to be “ready,” out of home care practitioners can be trained in the proactive motivational stance central to the Sleeping Dogs method and learn concrete strategies to introduce and integrate treatment towards the child (Dwyer et al., 2024). Moreover, results emphasize the importance of coordinated team-based collaboration around the child. Implementation efforts should therefore focus on improving interdisciplinary communication, aligning goals and responsibilities within the network, and out of home care organization and ensuring that all professionals share a coherent approach to motivating and supporting the child (Harris et al., 2025). Supervisors can facilitate this by helping practitioners reflect on their interactions, explore potential barriers, and apply these principles consistently.

Involving biological as well as foster parents in the therapeutic process seems to be crucial in motivating children for therapy, and makes it more likely that children can stay with- or return to live with their families (Mcllwaine et al., 2020). Practitioners may benefit from learning how to activate caregivers early on, explain trauma-related mechanisms in accessible terms, and coach them in providing emotional safety and predictability. Importantly, this also requires out of home care practitioners to deliberately invest time, attention, and effort in creating the space to involve the broader caregiving system. Engaging key figures in the child’s life is not merely an additional step in the process, but it demands a willingness to step beyond the individual therapeutic relationship, to reach out to the biological family, and to navigate complex family or network dynamics of intergenerational trauma (Harris et al., 2025). Building this confidence and professional courage should therefore be an explicit focus in training and supervision.

One area worth exploring further is to evaluate the implementation process of the Sleeping Dogs method in more detail, such as identifying all the steps and interventions that are provided to each child and the adherence to the method. The Sleeping Dogs method is an assessment-driven approach, with an emphasis on applying interventions in a customized flexible way that can be adapted to suit the individual needs of the child. This variability in interventions makes it difficult to generalize findings across all children receiving the intervention. However, since we did not look into variations between children, we do not know which characteristics of the children were associated with the duration of treatment, or positive outcomes. Future studies using a quantitative longitudinal design with a larger, more diverse group could focus more specifically on the adherence and (positive) completion of the Sleeping Dogs method, and could specify the use of therapeutic interactions and techniques within the Sleeping Dogs method.

## Conclusion

This study examined the experiences of both children and practitioners regarding the mechanisms behind the Sleeping Dogs method. This approach is tailored specifically to engage children with chronic trauma who are initially unable or unwilling to discuss their traumatic experiences and engage in trauma treatment. By providing motivational interventions, strengthening the child’s feelings of control and autonomy and connectedness, and involving the people around the child, including the parents, the foster parents and the care team, the Sleeping Dogs method helps children to become more receptive to trauma processing and trauma treatment. These results highlight the method’s potential as a valuable tool for working with a highly complex population, and underscore the need for further research to evaluate its impact and efficacy.

## Appendix A. Interview Guideline

“Sleeping Dogs is used with young people who are unable or unwilling to talk about their distressing or traumatic memories. Sleeping Dogs can help them eventually open up. This was the case with you as well. You have worked hard recently because you had painful memories of things you had experienced. You don’t have to tell me anything about what you went through, but I am curious about how you managed to start talking about those distressing memories.”

- Why did you not want to talk about your traumatic experiences? What did you find difficult about it?
- What helped you to talk about the distressing memories after the treatment?

*Safety*:

“Some children are unable or unwilling to talk because it makes them feel bad again or they feel unsafe. Other children are afraid of losing someone or being punished if they talk about the events.

Did this apply to you?

- No: Can you tell me more about this? How was this for you?
- I don’t know: Should I explain more?
- Yes.

Did the treatment help you with this?

- Yes: How did the treatment help you? Can you give an example?
- No: Why not?

*Daily Life*:

“We know that for some children, the distressing events can affect things in their daily lives. For example, going to school or sleeping.

Was this the case for you?

- No: Can you tell me more about this? Why was this not the case for you?
- I don’t know: Should I explain more?
- Yes.

Did the treatment help you with this?

- Yes: What was helpful for you (from the group, foster care worker, practitioner, etc.)? Why was it helpful? Can you give an example?
- No: Why did the treatment not help you?

*Attachment*:

“Some young people are afraid that it might be difficult for their parents or family to think back to the past. That is why they don’t want to talk about it themselves. They are somewhat protective of their parents or family. Other young people have no one to talk to about it. They are not sure if they can trust the group leaders or foster parents, or whether they can talk to them about it.

Was this the case for you?

- No: How was this for you? Why was this not the case for you?
- I don’t know: Should I explain more?
- Yes.

Did the treatment help with this?

- Yes: In what way did the treatment help you? How did you do it? What helped you?
- No: Why did the treatment not contribute to this?

Did you miss anything? Why did you miss that?”

*Emotion Regulation*:

“Almost every child who has experienced distressing events can suddenly become very angry, scared, or sad. Some young people are afraid that if they talk about it in therapy, they will become very angry, sad, or anxious.

Was that the case for you?

- No.
- Yes.

Did the treatment help with this?

- Yes: How did you manage to get better control over your emotions during the treatment? What was helpful? How do you use this, for example, at school, at home, or with friends?
- No: Can you explain why the treatment did not help with this?

Did you miss anything? Why did you miss that?”

*Cognitive Shift*:

“We sometimes see that children blame themselves for the distressing events. Some children are afraid that their father or mother will think they did something wrong.

Was this the case for you?

- No.
- Yes.

Did the treatment help with this?

- Yes: Was there something that helped you feel less ashamed so that you dared to do EMDR? Who did what? What was helpful? Can you give an example?
- No: Why did the treatment not contribute to this?

Did you miss anything? Why did you miss that?”

“We have just gone through various topics that made it easier for you to talk about the distressing events.

- Were there any other things besides what we just discussed that helped you to talk about the events?
- Do you have any tips for young people who are not yet able or willing to talk about their distressing memories? Or tips for practitioners working with Sleeping Dogs?
- What have I forgotten to ask? Do you want to add anything?”
